# Dual-Omics Profiling of Carotid Plaques Reveals Stage-Dependent Host–Microbiome Interaction Dynamics from Formation to Rupture

**DOI:** 10.3390/biomedicines14081708

**Published:** 2026-07-29

**Authors:** Shengnan Zhou, Ming Zhang, Shaobei Bai, Jinxiu Liu, Chunyan Zhang, Shuangli Mi, Jian Zhang

**Affiliations:** 1 Department of Nutrition and Microecology, TEDA International Cardiovascular Hospital, Tianjin 300457, China; 15102217771@163.com (S.Z.); zjx0113@sina.com (M.Z.); bbsspp@163.com (S.B.); grace.1225@163.com (J.L.); 13672059374@163.com (C.Z.); 2 Beijing Institute of Genomics, Chinese Academy of Sciences/China National Center for Bioinformation, Beijing 100101, China; 3 University of Chinese Academy of Sciences, Beijing 100049, China; 4 Beijing Key Laboratory of Intelligent Governance and Application of Biological Big Data, China National Center for Bioinformation, Beijing 100101, China; 5 Department of Molecular Pharmacology, National Clinical Research Center for Cancer, Key Laboratory of Cancer Prevention and Therapy, Tianjin, Tianjin’s Clinical Research Center for Cancer, Tianjin Medical University Cancer Institute & Hospital, Tianjin 300060, China

**Keywords:** carotid atherosclerosis, intraplaque microbiota, host–microbiome interactions, plaque rupture, transcriptomics, causal mediation analysis

## Abstract

**Background**: Carotid plaque rupture is a critical event in ischemic stroke, yet the potential involvement of the intraplaque microbiota across disease stages remains unclear. **Methods**: We performed dual-omics profiling by analyzing host transcriptomes and PathSeq-derived microbiomes from 48 human carotid RNA-seq specimens spanning early lesions (intimal thickening; n = 10), stable plaques (n = 20), and unstable plaques (n = 18). Host transcriptomes were profiled alongside intraplaque microbiomes extracted via the GATK PathSeq pipeline with rigorous in silico decontamination. We integrated differential expression analysis, microbial diversity metrics, and functional inference. Furthermore, an integrated machine learning approach (incorporating Boruta feature selection) was employed to identify exploratory cross-kingdom diagnostic biomarkers. **Results**: Microbial beta diversity diverged significantly across disease stages, accompanied by the progressive upregulation of 54 host genes critical for extracellular matrix remodeling and immune chemotaxis. Strikingly, despite the inherent noise and artifacts associated with low-biomass sequencing, we computationally detected the distinct enrichment of 21 bacterial taxa in unstable plaques, predominantly oral and gut mucosal pathobionts. Computationally inferred functional profiling revealed that these unstable plaque-associated microbiota were significantly linked to predicted cell death, IL-17, and HIF-1 signaling pathways and exhibited strong positive correlations with host matrix-degrading transcripts. Statistical modeling suggested associative links among specific microbial enrichment, host transcriptomic dysregulation, and plaque instability, highlighting concurrent biological cross-talk. Importantly, our integrated machine learning pipeline established a 14-feature cross-kingdom biomarker panel (10 host genes and 4 bacteria) that discriminated stable from unstable plaques (cross-validated AUC = 0.869). **Conclusions**: Intraplaque microbiome dynamics computationally associate with host transcriptomic alterations during carotid plaque evolution. This synergistic host–microbiome association provides a hypothesis-generating framework linking microbial dysbiosis to plaque destabilization, offering novel mechanistic insights and highlighting the exploratory cross-kingdom biomarker panel as a highly promising foundation for future experimental validation and stage-tailored clinical diagnostics.

## 1. Introduction

Carotid atherosclerotic plaque rupture accounts for 15–20% of ischemic strokes and represents a major unmet clinical challenge [1]. Plaque vulnerability is characterized by extracellular matrix (ECM) degradation, macrophage infiltration, smooth muscle cell apoptosis, and neovascularization [2,3]; yet the molecular factors associated with the transition from stable to rupture-prone lesions remain incompletely understood.

Recent discoveries have revealed diverse bacterial communities within atherosclerotic plaques. Sequencing studies have identified microbial taxa partially overlapping with the oral microbiota [4], and specific bacterial signatures are associated with symptomatic atherosclerosis [5]. Epidemiological evidence links periodontitis to carotid intima–media thickness and stroke risk [6], whereas circulating microbial metabolites such as trimethylamine N-oxide (TMAO) predict atherosclerotic plaque burden [7]. These observations implicate intraplaque microbiota as potential contributors to carotid atherosclerotic pathogenesis.

However, the functional associations of intraplaque microbial colonization and the associative links through which these communities interact with host vascular cells remain poorly defined. Traditional microbiome profiling approaches, while effective at cataloging microbial diversity, provide limited insight into host–microbiome crosstalk at the molecular level. Conversely, transcriptomic studies of atherosclerotic plaques have predominantly focused on host gene expression programs, overlooking the microbial dimension entirely. This analytical disconnect has precluded a systems-level understanding of how intraplaque bacterial abundances correlate with host inflammatory and remodeling responses that govern plaque progression and rupture. Moreover, whether host and microbial factors serve as concurrent potential contributors to disease progression across different stages of plaque evolution remains an open question with significant therapeutic implications.

Recent methodological advances have enabled the simultaneous extraction of host transcriptomic and microbial abundance profiles from RNA sequencing (RNA-seq) data [8]. The PathSeq algorithm, for instance, leverages non-human reads discarded during standard transcriptomic analyses to reconstruct microbial community composition, thereby enabling dual-omics profiling from a single sequencing experiment [8]. Specifically, total RNA-seq (rRNA-depleted bulk RNA-seq) offers a unique analytical advantage by circumventing poly-A selection bias, thereby allowing the unbiased, simultaneous capture of host mRNAs and microbial transcripts from the exact same physical tissue biopsy. This approach offers a cost-effective strategy for retrospectively mining publicly available RNA-seq datasets to investigate host–microbiome associations in archived clinical specimens. Furthermore, the application of statistical mediation analysis to such integrated datasets provides a framework for exploring potential associative links among microbiota, host gene expression, and disease outcomes.

In this study, we performed an integrative analysis of host transcriptomes and intraplaque microbiota using RNA-seq data from 48 human carotid artery specimens spanning three progressive disease stages: early lesions (diffuse intimal thickening), stable plaques, and unstable plaques. By systematically dissecting microbial community dynamics, host gene expression trajectories, and their associative links across disease progression, we sought to address three fundamental questions through a hypothesis-generating computational lens: (1) How does the intraplaque microbial community structure evolve during carotid atherosclerotic plaque progression? (2) Which host transcriptional programs are coordinately dysregulated with microbial compositional shifts? (3) How do host and microbial factors correlate with disease progression differently during plaque formation versus plaque rupture? Our findings reveal that plaque destabilization is associated with a coordinated multi-omics shift, wherein specific host transcriptomic alterations and the concomitant enrichment of mucosal-associated bacterial signatures correlate tightly with plaque vulnerability. Moreover, to translate these multi-omics insights into diagnostic utility, we utilized integrated machine learning with internal cross-validation to identify an exploratory 14-feature cross-kingdom biomarker panel (encompassing 10 host genes and 4 bacterial taxa), which demonstrated discriminatory power for identifying rupture-prone lesions. These computational insights highlight intraplaque microbial alterations as stage-dependent features of carotid atherosclerosis, providing a hypothesis-generating, data-driven framework for future experimental validation and the development of multi-omics diagnostic biomarkers.

## 2. Materials and Methods

### 2.1. Data Acquisition and Sample Classification

Transcriptomic datasets of human carotid artery plaques were obtained from the National Center for Biotechnology Information Gene Expression Omnibus (GEO) database (https://www.ncbi.nlm.nih.gov/geo, accessed on 15 May 2026). To ensure sufficient statistical power and biological representation, three independent bulk RNA-seq datasets (GSE120521, GSE198600, and GSE104140) were downloaded and integrated. Following manual curation of clinical phenotypes and pathological descriptions, 48 samples were retained and categorized into three progressive disease stages: (1) early lesion group (n = 10; described as “diffuse intimal thickening”); (2) stable plaque group (n = 20; described as “stable section of human atherosclerotic plaque”, “asymptomatic carotid atherosclerotic plaques”, or “fibroatheroma with calcification”); and (3) unstable plaque group (n = 18; described as “unstable section of human atherosclerotic plaque”, “symptomatic carotid atherosclerotic plaques”, or “fibroatheroma, noncalcified”). Detailed information, including clinical presentations (e.g., history of transient ischemic attack [TIA]/acute stroke), explicit RNA capture strategies and sequencing platforms for all participating cohorts, is provided in Table S1.

### 2.2. Host Transcriptome Processing and Differential Expression Analysis

Raw sequencing reads were subjected to quality control using FastQC (v0.11.9). High-quality reads were aligned to the human reference genome (GRCh38/hg38) using HISAT2 (v2.2.1) with default parameters. Gene-level read counts were quantified using HTSeq-count (v0.5.4) with the parameters “-s no -a 10”. To identify genes exhibiting dynamic expression changes across plaque progression, differential expression analysis was conducted using the edgeR package (v4.6.2) in R. Raw read counts were first normalized utilizing the trimmed mean of M-values (TMM) method implemented within edgeR to account for variations in library size. Differentially expressed genes (DEGs) were identified using a threshold of *p* < 0.05 and |fold change| > 1.6.

### 2.3. Microbial Profiling via the PathSeq Pipeline

To detect and quantify the intraplaque microbiome from host transcriptomic data, unmapped reads (i.e., reads that failed to align to the human genome) were processed using the PathSeq computational pipeline [8]. This subtraction-based approach involves sequential steps of stringent host sequence filtration, low-quality read removal, and low-complexity sequence filtering. The remaining non-host, high-quality reads (minimum post-trimming read length of 50 bp) were then aligned against a comprehensive reference database (GATK PathSeq bundle release 2017-12) containing bacterial, viral, and archaeal RefSeq genomes using the Burrows–Wheeler Aligner (BWA-MEM, v0.7.17) with a minimum alignment score of 30. Raw microbial read counts were calculated. The resulting taxonomic abundance matrices were filtered to require a minimum of 1 read per taxon per sample. Finally, the filtered counts were normalized to relative abundances for downstream ecological and taxonomic analyses. Given that the detection of microbial sequences from low-biomass human RNA-seq data remains technically challenging and highly susceptible to environmental contamination, mapping artifacts, and sequencing noise, all microbial findings derived from this computational approach were systematically treated as hypothesis-generating associations.

To mitigate the impact of environmental contamination, sequencing artifacts, and background noise, a stringent three-step filtering protocol was applied to the microbial profiles prior to any downstream analysis. First, to address the “kitome” and environmental contamination phenomena, a comprehensive blacklist of contaminant genera frequently detected in sequenced negative “blank” controls was compiled based on established guidelines [9,10]. Consequently, the following 84 taxa were systematically removed from our dataset: *Abiotrophia*, *Acidovorax*, *Acinetobacter*, *Aeromicrobium*, *Afipia*, *Aquabacterium*, *Arthrobacter*, *Asticcacaulis*, *Aurantimonas*, *Azoarcus*, *Azospira*, *Bacillus*, *Beijerinckia*, *Beutenbergia*, *Bosea*, *Bradyrhizobium*, *Brevibacillus*, *Brevibacterium*, *Brevundimonas*, *Brochothrix*, *Burkholderia*, *Caulobacter*, *Chryseobacterium*, *Comamonas*, *Corynebacterium*, *Craurococcus*, *Cupriavidus*, *Curtobacterium*, *Curvibacter*, *Deinococcus*, *Delftia*, *Devosia*, *Dietzia*, *Duganella*, *Dyadobacter*, *Enhydrobacter*, *Enterobacter*, *Escherichia*, *Facklamia*, *Flavobacterium*, *Geodermatophilus*, *Herbaspirillum*, *Hoeflea*, *Hydrotalea*, *Janibacter*, *Janthinobacterium*, *Kingella*, *Kocuria*, *Leptothrix*, *Limnobacter*, *Massilia*, *Mesorhizobium*, *Methylobacterium*, *Methylophilus*, *Methyloversatilis*, *Microbacterium*, *Micrococcus*, *Microlunatus*, *Nevskia*, *Niastella*, *Novosphingobium*, *Ochrobactrum*, *Olivibacter*, *Oxalobacter*, *Paenibacillus*, *Paracoccus*, *Patulibacter*, *Pedobacter*, *Pedomicrobium*, *Pelomonas*, *Phyllobacterium*, *Polaromonas*, *Propionibacterium*, *Pseudomonas*, *Pseudoxanthomonas*, *Psychrobacter*, *Ralstonia*, *Rhizobium*, *Rhodococcus*, *Roseomonas*, *Schlegelella, Sphingobium*, *Sphingomonas*, *Sphingopyxis*, *Stenotrophomonas*, *Streptococcus*, *Sulfuritalea*, *Tsukamurella*, *Undibacterium*, *Variovorax*, *Wautersiella*, and *Xanthomonas*. Second, a two-step statistical noise reduction was implemented: a prevalence filter retained only taxa present in at least 10% of the total samples (n ≥ 5) to ensure sufficient statistical power, and an abundance filter excluded taxa with a cumulative relative abundance across all samples of less than 10^−5^ to minimize index-hopping artifacts [11]. Finally, to maximize biological plausibility, the refined RNA-seq-derived genus-level profiles were cross-referenced with external, published 16S rRNA gene sequencing datasets (GSE308470) derived specifically from human atherosclerotic plaques [12]. Only microbial taxa concurrently detected in both our cohort and the 16S rRNA reference datasets were retained for ultimate analyses. The absolute counts and relative proportions of microbial reads recovered per sample across all cohorts are detailed in Table S2.

### 2.4. Data Integration and Modality-Specific Batch Effect Correction

Given that the integration of multiple independent datasets inevitably introduces non-biological technical variation, batch effect correction was implemented prior to downstream analyses. Recognizing the fundamental statistical differences between transcriptomic count data and compositional microbiome data, modality-specific correction strategies were employed. For the host transcriptomic matrix, batch effects across datasets were adjusted using ComBat-Seq from the sva package (v3.56.0) in R, which is specifically designed to handle the negative binomial distribution inherent in raw RNA-seq count data. For the microbial abundance matrix, which is characterized by zero inflation and compositional constraints, batch correction was performed utilizing MMUPHin (v1.22.0). This approach represents a gold standard methodology explicitly developed for the meta-analysis and batch adjustment of microbial community profiles. The effectiveness of batch effect removal for both host and microbial matrices was visually confirmed via principal component analysis (PCA) using the prcomp function, with the first two principal components plotted utilizing the ggbiplot package (v0.6.2). All subsequent statistical testing and feature selection were strictly performed on these corrected matrices.

### 2.5. Microbial Community Diversity and Taxonomic Analysis

Within-sample microbial richness and evenness (alpha diversity) were evaluated using the Shannon and Simpson indices via the vegan package (v2.7-2) in R. Differences in alpha diversity among the three groups were assessed using the Kruskal–Wallis test. Between-sample microbial community dissimilarity (beta diversity) was calculated based on the Bray–Curtis distance matrix and visualized by principal coordinate analysis (PCoA). The statistical significance of compositional differences across groups was determined using permutational multivariate analysis of variance (PERMANOVA) with 999 permutations. Differences in microbial taxon abundances across disease stages were initially evaluated using the Kruskal–Wallis test. For taxa exhibiting global significance, subsequent pairwise Wilcoxon rank-sum tests were performed. All *p* values were adjusted for multiple testing using the Benjamini–Hochberg FDR method.

### 2.6. Weighted Gene Co-Expression Network Analysis (WGCNA)

To identify clusters of highly correlated genes and explore their associations with plaque progression, WGCNA was performed using the WGCNA package (v1.74) in R. After filtering out genes with low variance, a scale-free topology fitting index (*R*^2^) and mean connectivity were calculated to determine the optimal soft-thresholding power (*β*). Using the selected power, an adjacency matrix was constructed and transformed into a topological overlap matrix (TOM) to measure network interconnectedness. Genes were clustered using average linkage hierarchical clustering based on TOM-based dissimilarity. Gene modules were identified using the dynamic tree cut algorithm. To pinpoint the most biologically relevant module for plaque vulnerability, the distribution of the 54 previously identified host DEGs across the generated modules was mapped. The module containing the highest concentration of target DEGs (e.g., the module eigengene 2 [ME2] module) was selected as the key host transcriptomic signature for downstream statistical mediation analysis.

### 2.7. Statistical Mediation Analysis

To investigate potential associative links among intraplaque microbiota composition, host gene expression, and disease status, statistical mediation analysis was performed using the mediation package (v4.5) in R. Based on the WGCNA results, we hypothesized an associative model wherein microbial dysbiosis is associated with plaque vulnerability and potentially linked to this process through specific host gene responses. Specifically, the following directional pathway was tested: bacterial abundance (Exposure, *X*) → host gene response (Mediator, *M*) → disease group (Outcome, *Y*). The host gene expression score serving as the mediator was derived from the target WGCNA module (i.e., the ME2 module, which encompassed the majority of the 54 crucial DEGs) utilizing gene set variation analysis (GSVA; GSVA package, v1.52.3). Two regression models were constructed: (1) a mediator model, in which the ME2 module score was regressed on bacterial abundance (*M* ~ *X*), and (2) an outcome model, in which disease status was regressed on both bacterial abundance and the ME2 module score (*Y* ~ *X* + *M*). The total effect was decomposed into the average causal mediation effect (ACME; representing the indirect statistical association mediated through the host gene expression score) and the average direct effect (ADE; representing the unmediated association between bacterial abundance and disease). For the models, statistical significance was assessed using nonparametric bootstrapping with 1000 resampling iterations to compute percentile-based 95% confidence intervals (CIs). To account for multiple testing across the mediation models, the Benjamini–Hochberg FDR correction was applied. Both nominal and FDR-adjusted *p* values for the ACME and ADE are detailed in Table S3.

### 2.8. Functional Prediction and Pathway Enrichment

To interpret the biological significance of the identified host DEGs, Gene Ontology (GO) enrichment analysis was performed using the online DAVID tools (https://davidbioinformatics.nih.gov/). The analysis specifically focused on the GO Biological Process (BP) category. Significantly enriched terms were identified using the hypergeometric test, with *p* < 0.05 considered statistically significant.

For microbial communities, functional potential was inferred based on Kyoto Encyclopedia of Genes and Genomes (KEGG) pathway analysis of the taxonomic composition. Only taxa resolved to the species level were retained. Each species name was mapped to a KEGG organism code (https://rest.kegg.jp/list/{organism_code}, accessed on 15 May 2026). For species that could not be directly mapped to a KEGG organism code, the phylogenetically closest congeneric species was identified using the NCBI Taxonomy database (https://www.ncbi.nlm.nih.gov/taxonomy, accessed on 15 May 2026) and used as a surrogate for functional annotation. For each successfully mapped organism (either directly or via surrogate assignment), the full set of KEGG Orthology (KO) identifiers was retrieved via the KEGG API (https://rest.kegg.jp/link/ko/{organism_code}, accessed on 15 May 2026). KO identifiers were then mapped to KEGG metabolic pathways (https://rest.kegg.jp/link/pathway/ko, accessed on 15 May 2026). Only reference pathways (prefixed with “map”) were retained for downstream analysis. The predicted functional profile (pathway-level abundance/score) for each sample was calculated as follows:

$${Pathway}_{p}\;=\;\sum_{i\;=\;1}^{n}\;(A_{i}\times C_{ip})$$

where *A_i_* is the relative abundance of species *i* in a given sample, and *C_ip_* is the total count of KOs assigned to pathway *p* that are present in the reference genome of species *i* (or its assigned surrogate). Pathway names were annotated using the KEGG pathway list (https://rest.kegg.jp/list/pathway, accessed on 15 May 2026). Finally, statistical differences in pathway abundances between the stable and unstable groups were evaluated using the Wilcoxon rank-sum test. To account for multiple testing, *p* values were adjusted using the Benjamini–Hochberg FDR method.

### 2.9. Integrated Feature Prioritization Analysis

To prioritize candidate biomarkers from the integrated host–microbe dataset, we employed the Boruta algorithm, a wrapper-based feature selection method implemented in the Boruta package (v9.0.0) in R. The combined feature matrix comprised 54 progressively upregulated DEGs and 21 unstable plaque-enriched bacterial taxa identified from differential abundance analysis. Boruta iteratively evaluates feature importance by comparing each original feature against randomly permuted “shadow” attributes using a random forest classifier, retaining only those features whose importance is statistically confirmed to exceed that of the best-performing shadow feature (maxRuns = 500, seed = 42). Features classified as “confirmed” by the Boruta algorithm were subsequently ranked by the mean decrease in Gini (MDG) impurity derived from a random forest model (randomForest package, v4.7.1.2; ntree = 1000) to quantify their relative discriminatory contributions. To evaluate the collective discriminatory capacity of the Boruta-confirmed features, a random forest classifier was constructed to distinguish stable (n = 20) from unstable plaques (n = 18). To evaluate model performance while mitigating overfitting, we utilized a 5-fold cross-validation framework via the caret package (v7.0.1). Within this framework, down-sampling was incorporated during the resampling process (caret: trainControl, sampling = “down”) to ensure balanced and unbiased class learning. The diagnostic efficacy of the cross-validated model was subsequently assessed using receiver operating characteristic (ROC) analysis, with the area under the curve (AUC) and 95% CIs computed using the pROC package (v1.19.0.1).

### 2.10. Single-Cell RNA Sequencing Validation Analysis

To independently validate the expression patterns of the key host genes identified by our machine learning panel at single-cell resolution, we obtained a published scRNA-seq dataset of human carotid plaques from the GEO database (GSE253903). This validation cohort comprises 12 samples, including 6 stable and 6 unstable plaques. Raw count matrices were processed utilizing the Seurat package (v5.5.0) in R. A rigorous quality control protocol was applied to filter out low-quality cells, empty droplets, and multiplets. Specifically, cells were retained for downstream analysis only if they met the following criteria: expressing between 200 and 5000 unique features (genes) and possessing a mitochondrial gene expression fraction of less than 15%. The filtered data were log-normalized, and the top 2000 highly variable genes were identified using the “vst” method. Following data scaling, PCA was performed. The first 20 principal components (PCs) were utilized for graph-based clustering (FindClusters, resolution = 0.5) and uniform manifold approximation and projection (UMAP) dimensionality reduction for 2D visualization. Cell clusters were systematically annotated into major plaque-resident lineages (including macrophages, T cells, endothelial cells, fibroblasts, and smooth muscle cells) based on the robust expression of established canonical marker genes (e.g., *CD68*, *CD14*, *MYH11*, *ACTA2*, *VWF*, *PECAM1*, *CD3D*, *CD2*, *DCN*, and *COL1A1*). Finally, to validate the differential expression of our target host genes between unstable and stable plaques within specific cell subpopulations, the Wilcoxon rank-sum test was conducted using Seurat’s FindMarkers function. The percentage of cells expressing the target genes and their average expression levels were visualized via dot plots. Statistical significance for differential expression at the single-cell level was annotated based on standard thresholds (* *p* < 0.05, ** *p* < 0.01, *** *p* < 0.001, ns = not significant).

### 2.11. Statistical Analysis

All statistical analyses and visualizations were performed using R software (v4.4.1). Continuous variables were presented as the mean ± standard error of the mean (SEM) or the median with interquartile range (IQR), depending on data distribution as assessed by the Shapiro–Wilk test. To control for false positives in high-throughput analyses, the Benjamini–Hochberg FDR method was applied to all multiple comparisons. Unless otherwise specified, a two-sided *p* < 0.05 and an FDR < 0.05 were considered statistically significant.

## 3. Results

### 3.1. Integrated Multi-Cohort Workflow and Microbial Community Structure Across Disease Stages

**Figure 1:**
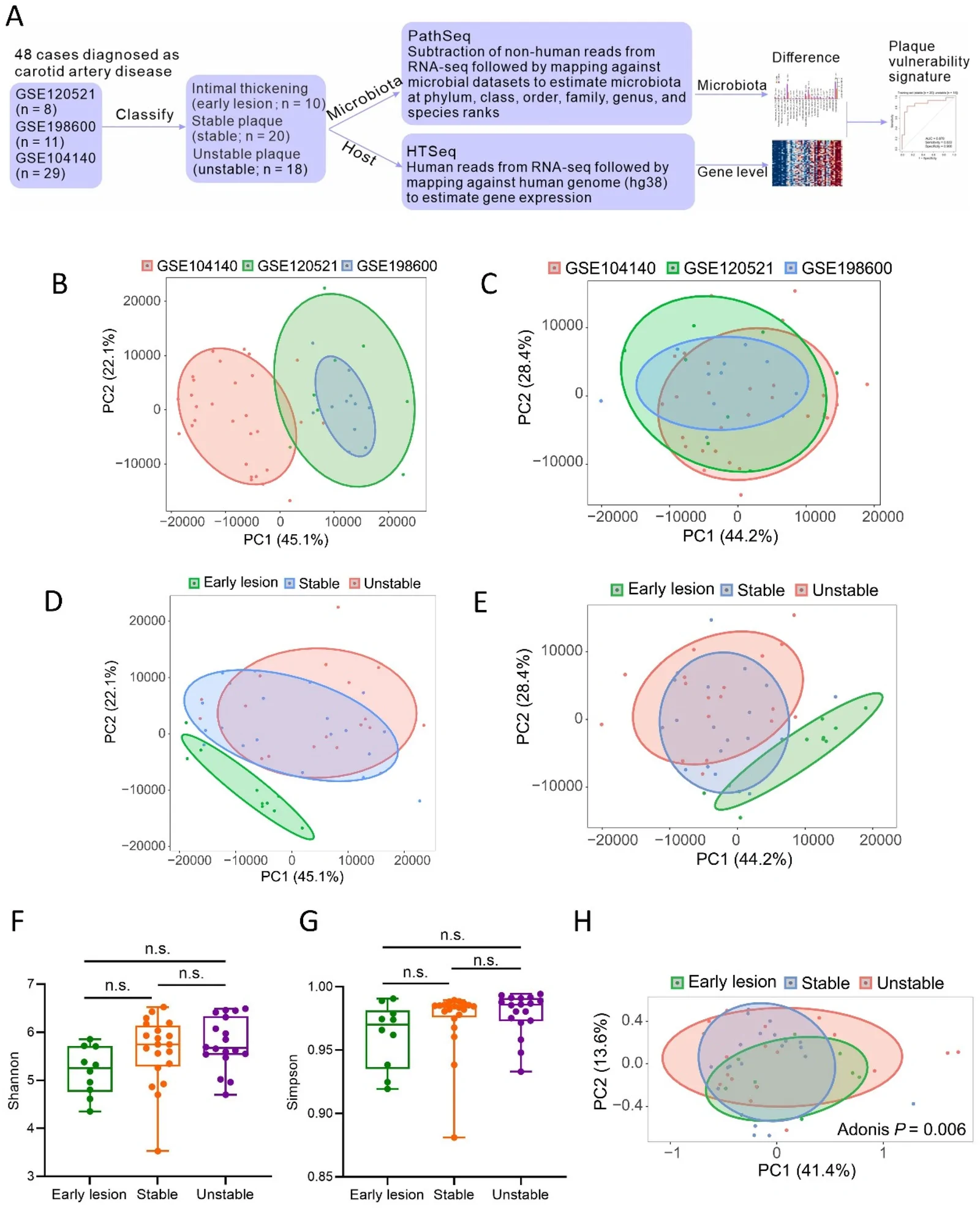
Study design and principal component analysis of transcriptomic and microbiome profiles in carotid artery disease. (**A**) Schematic overview of the study workflow. RNA-seq data from 48 patients with carotid artery disease were obtained from three GEO datasets (GSE120521, n = 8; GSE198600, n = 11; GSE104140, n = 29). Patients were classified into three groups: intimal thickening (Early lesion, n = 10), stable plaque (Stable, n = 20), and unstable plaque (Unstable, n = 18). Non-human reads were processed using PathSeq to estimate the microbiota composition at the genus and species levels, while human reads were mapped to the human reference genome (hg38) and quantified using HTSeq. Differential analyses were performed on both microbial abundance and host gene expression. These differences were ultimately utilized to construct and evaluate a “plaque vulnerability signature” for distinguishing plaque stability, as demonstrated by the ROC analysis. (**B**) PCA of gene expression and microbiome profiles colored by dataset origin (GSE104140, GSE120521, and GSE198600) before batch effect correction. PC1 and PC2 explain 45.1% and 22.1% of the total variance, respectively. (**C**) PCA of gene expression and microbiome profiles colored by dataset origin after batch effect correction. PC1 and PC2 explain 44.2% and 28.4% of the total variance, respectively. (**D**) PCA of gene expression and microbiome profiles colored by plaque type (Early lesion, Stable, and Unstable) before correction. PC1 and PC2 explain 45.1% and 22.1% of the total variance, respectively. (**E**) PCA of gene expression and microbiome profiles colored by plaque type after correction. PC1 and PC2 explain 44.2% and 28.4% of the total variance, respectively. (**F**) Shannon diversity index of microbial communities across the three plaque groups. Boxes represent the IQR, center lines indicate the median, and whiskers extend to 1.5× IQR. (**G**) Simpson diversity index of microbial communities across the three plaque groups. No statistically significant differences were observed for either alpha-diversity index. (**H**) PCoA plot based on Bray–Curtis dissimilarity demonstrating significant separation of samples by plaque type. Statistical significance was assessed by PERMANOVA (*p* = 0.006). PCA, principal component analysis; PCoA, principal coordinate analysis; IQR, interquartile range; n.s., not significant; PERMANOVA, permutational multivariate analysis of variance.

The bioinformatics workflow of our integrative transcriptomic and microbial profiling is illustrated in Figure 1A. Briefly, host gene expression was quantified from human-aligned reads using HTSeq, while microbial abundance profiles were simultaneously extracted from unmapped (non-human) reads using the PathSeq algorithm. To mitigate background noise and sequencing artifacts, we applied a stringent three-step decontamination strategy: (1) filtering against a predefined blacklist of 84 canonical contaminant taxa; (2) imposing statistical thresholds of ≥10% prevalence (n ≥ 5) and a cumulative abundance of ≥1 × 10^−5^; and (3) a rigorous fidelity check that exclusively retained microbial taxa concurrently validated in external, plaque-specific 16S rRNA benchmark datasets [12]. The paired host and microbial profiles were merged and consolidated across three independent RNA-seq datasets (GSE120521, GSE198600, and GSE104140), yielding a final cohort of 48 specimens. These samples spanned three progressive pathological stages: early lesions (diffuse intimal thickening, n = 10), stable plaques (representing clinically stable/asymptomatic disease, n = 20), and unstable plaques (representing clinically unstable disease, frequently associated with events such as TIA or acute stroke, n = 18).

To ensure robust downstream analyses, non-biological batch effects inherent to multi-cohort integration were eliminated using modality-specific strategies. ComBat-Seq was applied to the host count matrix to preserve its negative binomial distribution, whereas MMUPHin was deployed for the zero-inflated, compositional microbial matrix. PCA confirmed the successful removal of dataset-driven technical variation (Figure 1B,C). Importantly, re-projection of the batch-corrected data revealed a progressive transitional overlap among the three disease stages, indicating that the retained variance successfully captures the gradual, step-wise progression of the disease (Figure 1D,E).

We next evaluated intraplaque microbial community diversity. Alpha diversity analyses (Shannon and Simpson indices) revealed no statistically significant differences among the three groups (Figure 1F,G), suggesting that overall species richness and evenness remained broadly stable during plaque evolution. In striking contrast, beta diversity analysis based on Bray–Curtis dissimilarity (PERMANOVA, 999 permutations) demonstrated highly significant compositional divergence across disease stages (*p* = 0.006; Figure 1H). This dissociation between preserved alpha diversity and marked beta diversity shifts indicates that plaque progression towards rupture is associated with a systematic reorganization of microbial community architecture, rather than a mere expansion or contraction of the total microbial load.

### 3.2. Taxonomic Profiling Reveals Progressive Microbial Community Remodeling Across Disease Stages

**Figure 2:**
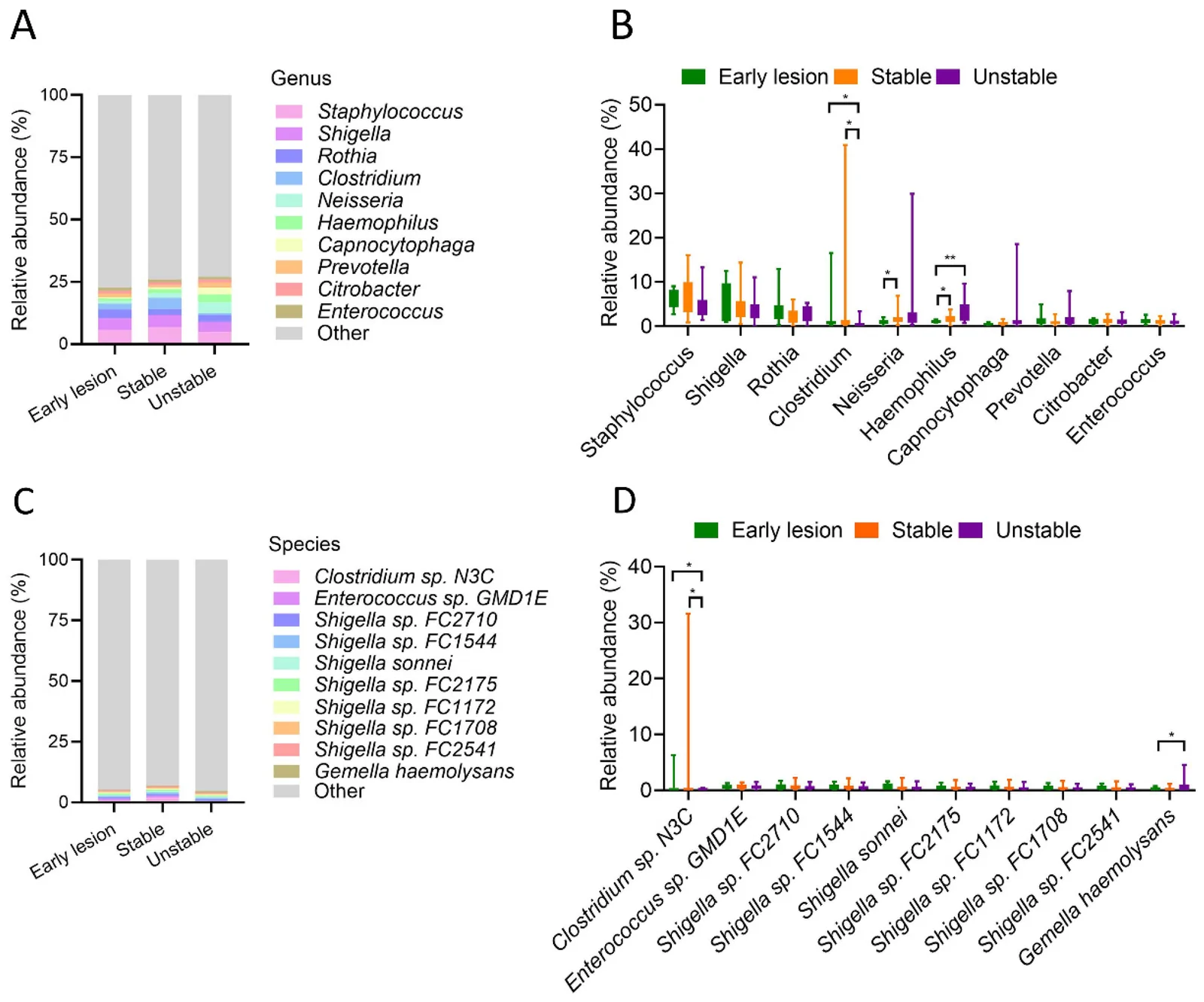
Microbiota composition at the genus and species taxonomic levels across carotid plaque groups. Microbial community composition was compared among the early lesion (intimal thickening, n = 10), stable plaque (n = 20), and unstable plaque (n = 18) groups at the genus and species levels. (**A**) Stacked bar chart showing the mean relative abundance of the ten most abundant bacterial genera across the three groups. (**B**) Box plots comparing the relative abundance of individual genera among the three groups. (**C**) Stacked bar chart showing the mean relative abundance of the ten most abundant bacterial species across the three groups. (**D**) Box plots comparing the relative abundance of individual species among the early lesion, stable, and unstable groups. In all box plots (**B**,**D**), boxes represent the IQR, center lines indicate the median, and whiskers extend to 1.5× IQR. Statistical significance was determined by pairwise Wilcoxon rank-sum tests. Statistical significance is denoted by asterisks (* *p* < 0.05; ** *p* < 0.01).

To delineate the specific compositional shifts underlying the observed beta diversity differences, we examined taxonomic profiles at finer resolution using the rigorously decontaminated microbial matrix. At the genus level, the intraplaque microbiota across all stages was predominantly composed of *Staphylococcus*, *Shigella*, *Rothia*, *Clostridium*, *Neisseria*, and *Haemophilus* (Figure 2A). In contrast to the preservation of broad alpha diversity, assessment of relative abundances revealed distinct, stage-specific microbial dynamics (Figure 2B). Notably, *Clostridium* exhibited a transient yet highly significant enrichment specifically within the stable plaque stage when compared to both early lesions (*p* < 0.05) and unstable plaques (*p* < 0.05). Similarly, *Neisseria* was significantly elevated in stable plaques relative to early lesions (*p* < 0.05). Conversely, *Haemophilus* displayed accumulation during the advanced disease phase, showing significantly higher relative abundance in the unstable group compared to both stable plaques (*p* < 0.05) and early lesions (*p* < 0.01) (Figure 2B).

Species-level profiling further refined these stage-specific signatures. The microbial community was primarily dominated by species such as *Clostridium* sp. *N3C*, *Enterococcus* sp. *GMD1E*, and multiple *Shigella* species (Figure 2C). Mirroring the genus-level dynamics, *Clostridium* sp. *N3C* characterized the distinct signature of the stable phase, exhibiting a significantly higher relative abundance in stable plaques compared to the other two stages (both *p* < 0.05) (Figure 2D). Importantly, *Gemella haemolysans* emerged as a prominent indicator of plaque destabilization, demonstrating a significantly elevated relative abundance in the unstable group compared to early lesions (*p* < 0.05) (Figure 2D). It is critical to note that the detection of these specific advanced-stage taxa (such as *Haemophilus* and *Gemella haemolysans*) survived our stringent three-tier decontamination pipeline and external 16S cross-referencing. Together, these data demonstrate that plaque progression is not a uniform process of microbial accumulation but rather a trajectory of dynamic, stage-specific succession.

### 3.3. Host Transcriptomic Profiling Identifies ECM Remodeling and Immune-Chemotactic Signatures Linked to Plaque Progression

**Figure 3:**
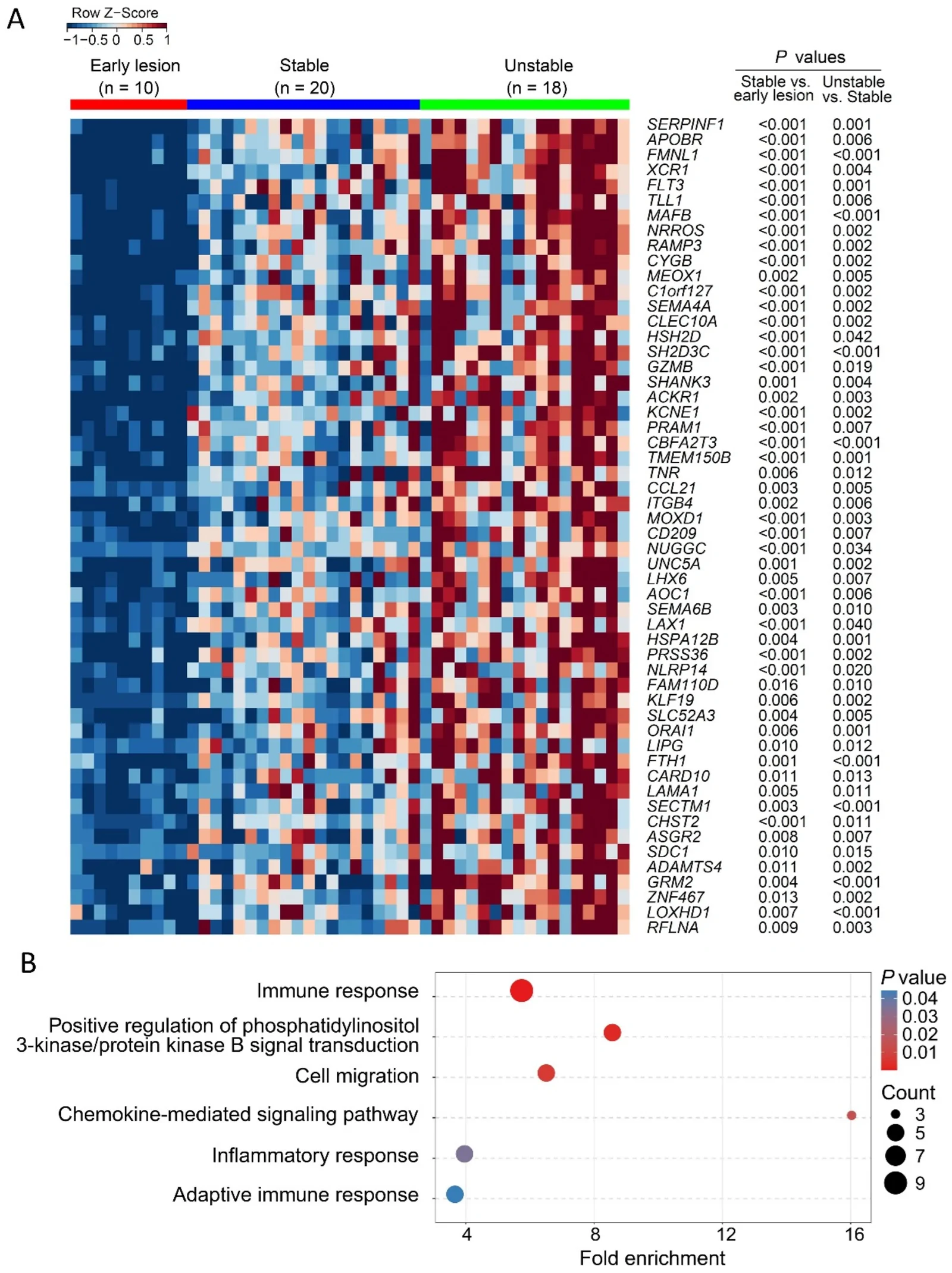
Differentially expressed genes and functional enrichment analysis across carotid plaque stages. (**A**) Heatmap displaying the expression patterns of 54 differentially expressed genes across the early lesion (n = 10), stable (n = 20), and unstable (n = 18) plaque groups. Expression values are presented as row-normalized Z-scores, with red indicating relatively high expression and blue indicating relatively low expression. The table on the right shows *p* values for pairwise comparisons between groups (stable versus early lesion, and unstable versus stable). All listed genes achieved statistical significance (*p* < 0.05) across both comparisons. (**B**) GO Biological Process enrichment analysis of the 54 differentially expressed genes. The x-axis represents fold enrichment, and the y-axis lists the enriched GO terms. Dot size corresponds to the number of genes associated with each term, and dot color represents the statistical significance (*p* value), with darker red indicating greater significance. GO, Gene Ontology.

Parallel to the microbial compositional shifts, we sought to identify host genes whose expression patterns dynamically tracked disease severity. Employing rigorous statistical criteria (pairwise comparisons across all three groups: *p* < 0.05; |fold change| > 1.6), we identified 54 genes that exhibited continuous, step-wise upregulation from the early lesion stage through stable plaque formation to eventual rupture, whereas no genes displayed sustained downregulation across this disease continuum (Figure 3A). To rule out the possibility that apparent stage-specific effects were confounded by dataset-of-origin effects, we performed a sensitivity analysis restricted exclusively to the largest single dataset (GSE104140). Importantly, the core host transcriptomic signature was successfully replicated within this single technical context, with ≥33% feature overlap consistently retained even when the GSE104140 dataset was evaluated in isolation (Table S4). Given the central role of ECM dysregulation in both plaque formation and destabilization [13], we systematically annotated these progressively upregulated genes using Naba’s matrisome classification criteria [14]. This targeted analysis successfully mapped 11 DEGs to the intraplaque matrisome network. Specifically, *LAMA1* and *TNR* were identified as ECM glycoproteins, directly reflecting structural alterations of the lesion scaffold. Crucially, a distinct cluster emerged as ECM regulators, including *ADAMTS4*, *SERPINF1*, and *TLL1*. The concerted upregulation of these proteases and enzymatic modulators implies an escalating state of proteolytic degradation and ECM turnover. Furthermore, the matrisome profile was populated by ECM-affiliated proteins (*CD209*, *CLEC10A*, *SDC1*, *SEMA4A*, and *SEMA6B*) and the secreted factor *CCL21*, dynamically mediating inflammatory cell adhesion, retention, and signaling within the microenvironment [15,16]. Collectively, this precise matrisome mapping reveals that progressive plaque evolution is closely associated with a coordinated upregulation of ECM-degrading machinery, simultaneously coupled with structural modifications that facilitate massive inflammatory cell infiltration.

Functional enrichment analysis of these 54 progressively upregulated DEGs confirmed the systemic activation of host inflammatory programs and cellular migration processes. The significantly enriched GO BP terms were heavily dominated by immune activation, specifically “immune response,” “inflammatory response,” and “adaptive immune response” (Figure 3B). Parallel to these broad immune signatures, processes orchestrating the physical recruitment of immune cells were highly enriched, notably the “chemokine-mediated signaling pathway” and “cell migration” (Figure 3B). Additionally, pivotal intracellular signaling cascades implicated in macrophage activation and smooth muscle cell phenotypic switching were significantly enriched. This prominently features the “positive regulation of phosphatidylinositol 3-kinase/protein kinase B (PI3K/Akt) signal transduction” pathway, which is an established factor in plaque progression, cell survival, and instability [17] (Figure 3B). Together, these transcriptomic signatures and functional enrichment data delineate a clear mechanistic trajectory: escalating chemokine signaling and ECM remodeling accompany continuous inflammatory cell migration into the plaque, while PI3K/Akt activation sustains their pathological persistence, ultimately culminating in structural rupture.

### 3.4. Microbial Enrichment and Functional Shifts During the Transition to Plaque Vulnerability

**Figure 4:**
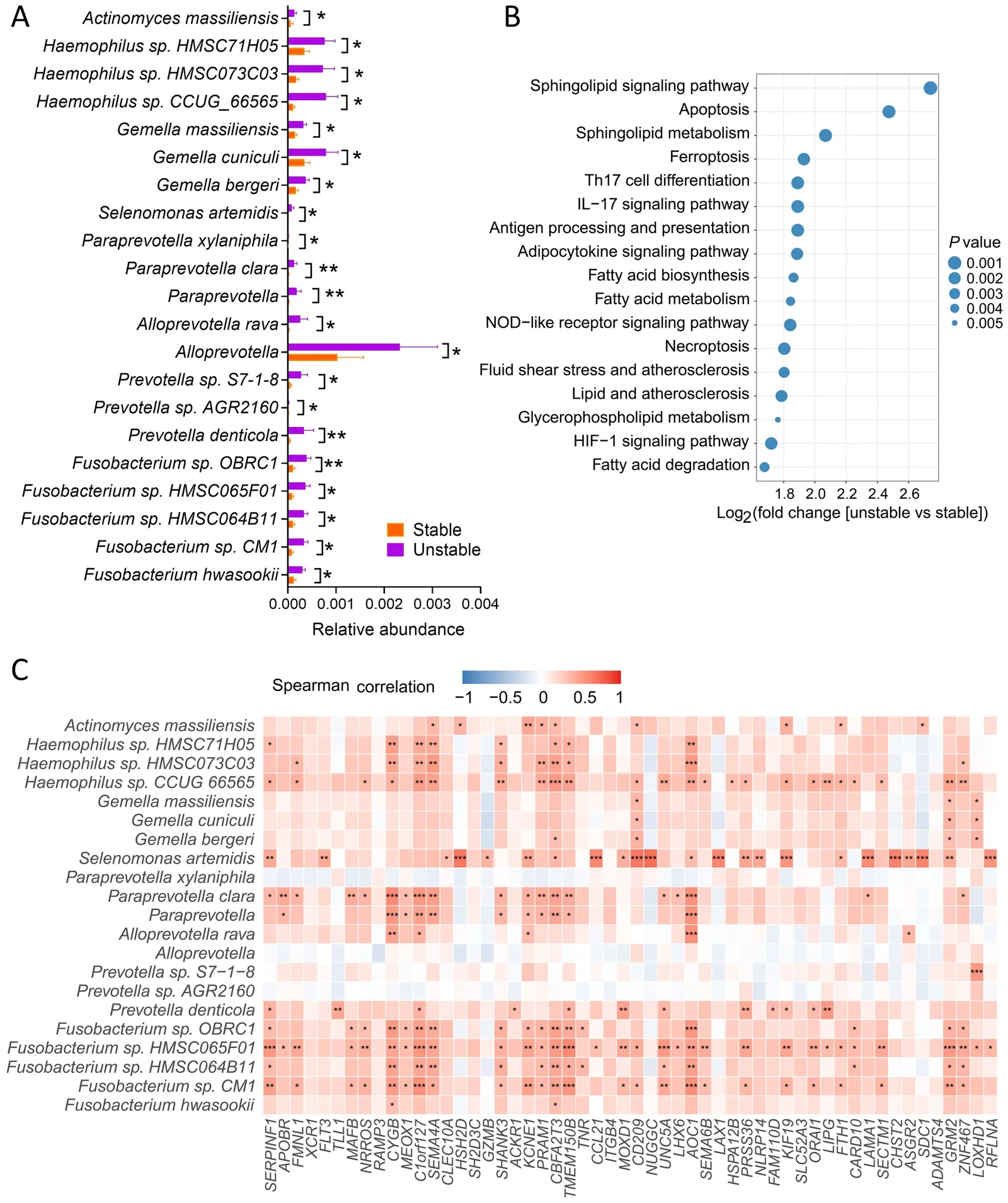
Unstable plaque-associated microbiota enrichment and metabolic pathway activation in unstable carotid plaques. (**A**) Relative abundance of bacterial taxa significantly enriched in unstable plaques compared with stable plaques. Data are presented as the mean ± SEM. (**B**) KEGG pathway analysis shows significantly enriched pathways upregulated in unstable plaques relative to stable plaques (*p* < 0.05). The x-axis represents the log_2_(fold change) between unstable and stable plaques, while the size of each circle corresponds to the *p* value, with larger circles indicating greater statistical significance (smaller *p* values). (**C**) Heatmap of Spearman correlation coefficients between the 54 progressively upregulated host differentially expressed genes (columns) and the 21 unstable plaque-enriched bacterial taxa (rows). Positive correlations are shown in red and negative correlations in blue. Statistical significance is denoted by asterisks (* *p* < 0.05; ** *p* < 0.01; *** *p* < 0.001). SEM, standard error of the mean; KEGG, Kyoto Encyclopedia of Genes and Genomes.

To identify specific microbial signatures associated with the critical transition from stable lesions to unstable, rupture-prone plaques, we performed differential abundance analysis comparing these two advanced disease stages. In stark contrast to earlier developmental stages, this analysis revealed a unidirectional expansion of the microbial community in unstable plaques. We identified 21 specific bacterial taxa (at the genus and species levels) that were significantly enriched in unstable lesions relative to stable plaques, whereas no taxa were significantly depleted (Figure 4A). This unstable plaque-associated consortium is notably dominated by classical oral and gut commensals/pathobionts, including multiple species within the genera *Fusobacterium*, *Haemophilus*, Actinomyces, *Prevotella*, *Alloprevotella*, *Paraprevotella*, *Gemella*, and *Selenomonas* (Figure 4A). In the context of our cross-sectional data, the detection of these predominantly anaerobic taxa in vulnerable vascular tissue represents hypothesis-generating associations that reinforce the concept of a potential mucocutaneous–vascular translocation axis during late-stage atherosclerosis.

To explore the speculative mechanistic plausibility of the association between these enriched pathobionts and plaque destabilization, we assessed their taxonomy-based predicted KEGG functional potential. The predicted pathways distinguishing the unstable plaque-enriched microbiota aligned strikingly with known markers of advanced plaque vulnerability (Figure 4B). Specifically, the taxonomy-based computational prediction of “apoptosis,” “ferroptosis,” and “necroptosis” pathways suggests a potential microbial amplification of programmed cell death cascades. Furthermore, a pronounced immunological shift was predicted, characterized by the enrichment of the “IL-17 signaling pathway,” “Th17 cell differentiation,” and “NOD-like receptor signaling pathway”. Additionally, alterations in “sphingolipid signaling/metabolism” and “HIF-1 signaling” suggest possible local lipid handling and hypoxia-driven responses (Figure 4B). It is crucial to note that these functional profiles represent inferred genomic capacity rather than measured in vivo activity.

Having established distinct host transcriptomic (54 progressively upregulated DEGs) and microbial (21 unstable plaque-enriched taxa) signatures, we next characterized their interaction dynamics during plaque destabilization. Spearman correlation analysis revealed a pervasive positive co-expression module: the unstable plaque-enriched bacterial taxa exhibited overwhelmingly positive correlations with the host DEGs implicated in ECM remodeling, immune chemotaxis, and cellular migration (Figure 4C). This positive bidirectional relationship suggests that microbial accumulation and host inflammatory/matrix-degrading responses are statistically coupled during the critical window of plaque instability.

### 3.5. Co-Expression Dynamics and Causal Mediation of Host–Microbiome Interactions

**Figure 5:**
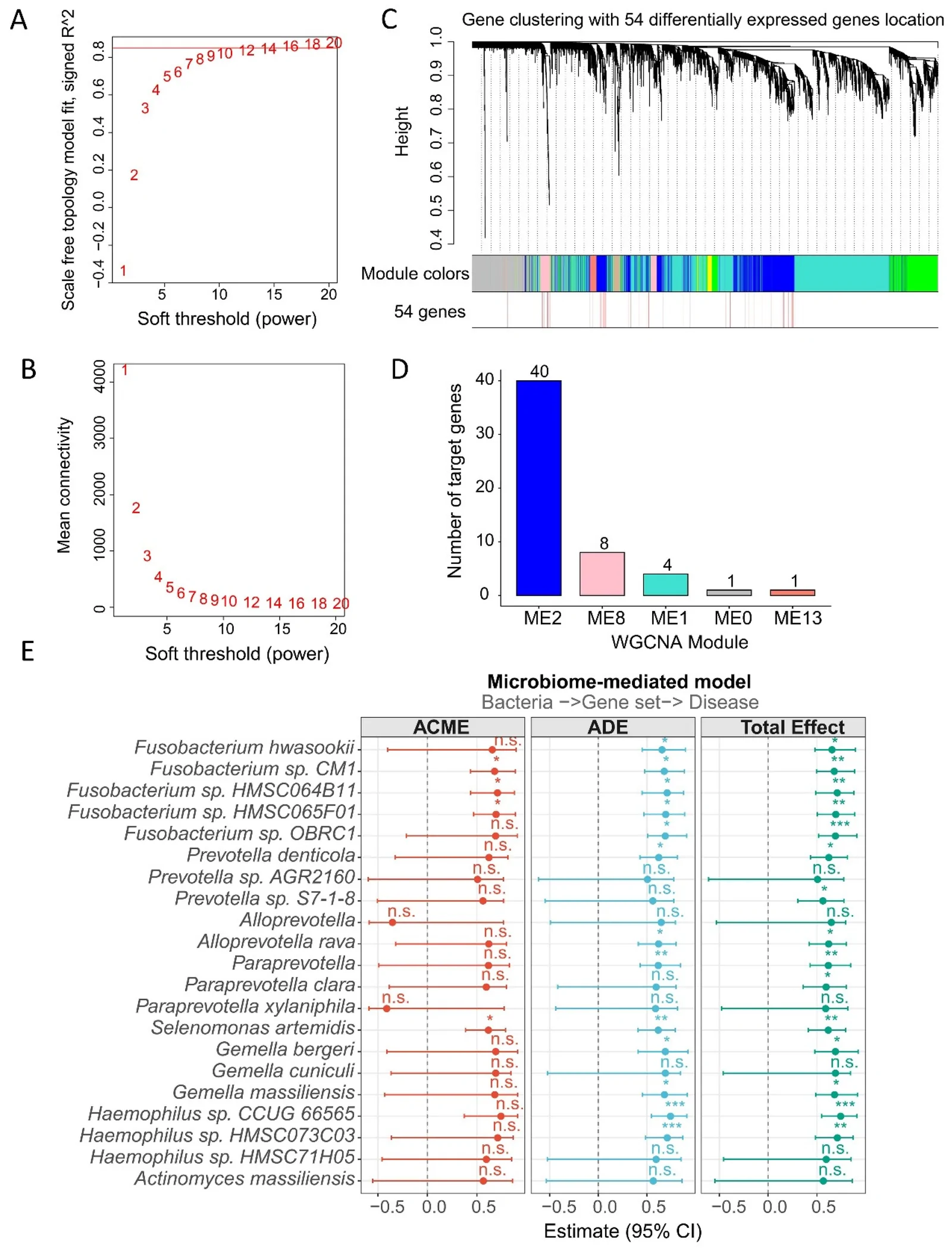
Identification of crucial gene modules via WGCNA and microbiome-mediated causal analysis. (**A**,**B**) Determination of the optimal soft-thresholding power (β) in WGCNA. (**A**) Analysis of the scale-free topology model fit index (*R*^2^) for various soft-thresholding powers. The red line represents the threshold for a scale-free network. (**B**) Analysis of the mean connectivity for various soft-thresholding powers. (**C**) Hierarchical clustering dendrogram of genes based on topological overlap. The first color band (“module colors”) indicates the assigned WGCNA module for each gene. The second band (“54 genes”) illustrates the distribution of the 54 identified differentially expressed genes across the hierarchical tree. (**D**) Bar chart showing the distribution counts of the 54 genes within the identified WGCNA modules. The blue module (ME2) shows the most significant enrichment, containing 40 target genes. (**E**) Forest plot detailing the results of the causal mediation analysis based on the hypothesized model (Bacteria → ME2 module → Disease). The plot displays the estimated average causal mediation effect (ACME, red, representing the indirect effect mediated by the gene set), average direct effect (ADE, blue, representing the direct effect not mediated through the ME2 module), and total effect (green), with their 95% confidence intervals, for selected microbiome species. Statistical significance is denoted by asterisks (* *p* < 0.05, ** *p* < 0.01, *** *p* < 0.001; n.s., not significant). WGCNA, weighted gene co-expression network analysis.

To formally interrogate the directional architecture of this host–microbe co-variation, we performed causal mediation analysis. To avoid the circular logic of using outcome-defined gene signatures directly as mediators, we employed WGCNA (Figure 5A,B). Mapping the 54 critical DEGs onto the generated unsupervised network revealed that the vast majority of these genes (40 out of 54) were highly concentrated within a single specific module, designated as ME2 (Figure 5C,D). Consequently, ME2 was selected as the unbiased surrogate mediator representing the host’s vulnerability-associated transcriptomic program.

Using a non-parametric bootstrap approach (1000 iterations), we evaluated a microbiome-mediated causal model (bacteria → host ME2 → disease status). The analysis demonstrated that the total effect of microbial enrichment on unstable plaque status was statistically significant for the majority of the tested taxa (Figure 5E). Interestingly, the ACME, representing the impact of bacteria acting through the host ME2 gene module, was largely non-significant for most taxa, with only marginal mediation observed for isolated strains like *Fusobacterium* sp. *CM1*, *Fusobacterium* sp. *HMSC064B11*, *Fusobacterium* sp. *HMSC065F01*, and *Selenomonas artemidis* (Figure 5E). Conversely, the ADE remained statistically significant across numerous key taxa. Guided by our statistical power analysis (detailed in the Methods), we recognize that the non-significant ACME observed here likely reflects insufficient statistical power inherent to the limited sample size of the stable-versus-unstable contrast, rather than true biological independence. Therefore, we explicitly refrain from describing the host and microbial factors as operating through “independent” or “parallel” pathways. Instead, the persistent significance of the ADE suggests that microbial enrichment is significantly associated with plaque destabilization alongside host transcriptomic changes. Given the cross-sectional nature of our study, these findings are explicitly reframed as hypothesis-generating associations regarding potential host–microbiome interactions rather than proof of definitive causal determinants.

### 3.6. Integrated Feature Analysis Identifies a Host–Microbiome Biomarker Panel for Plaque Vulnerability

**Figure 6:**
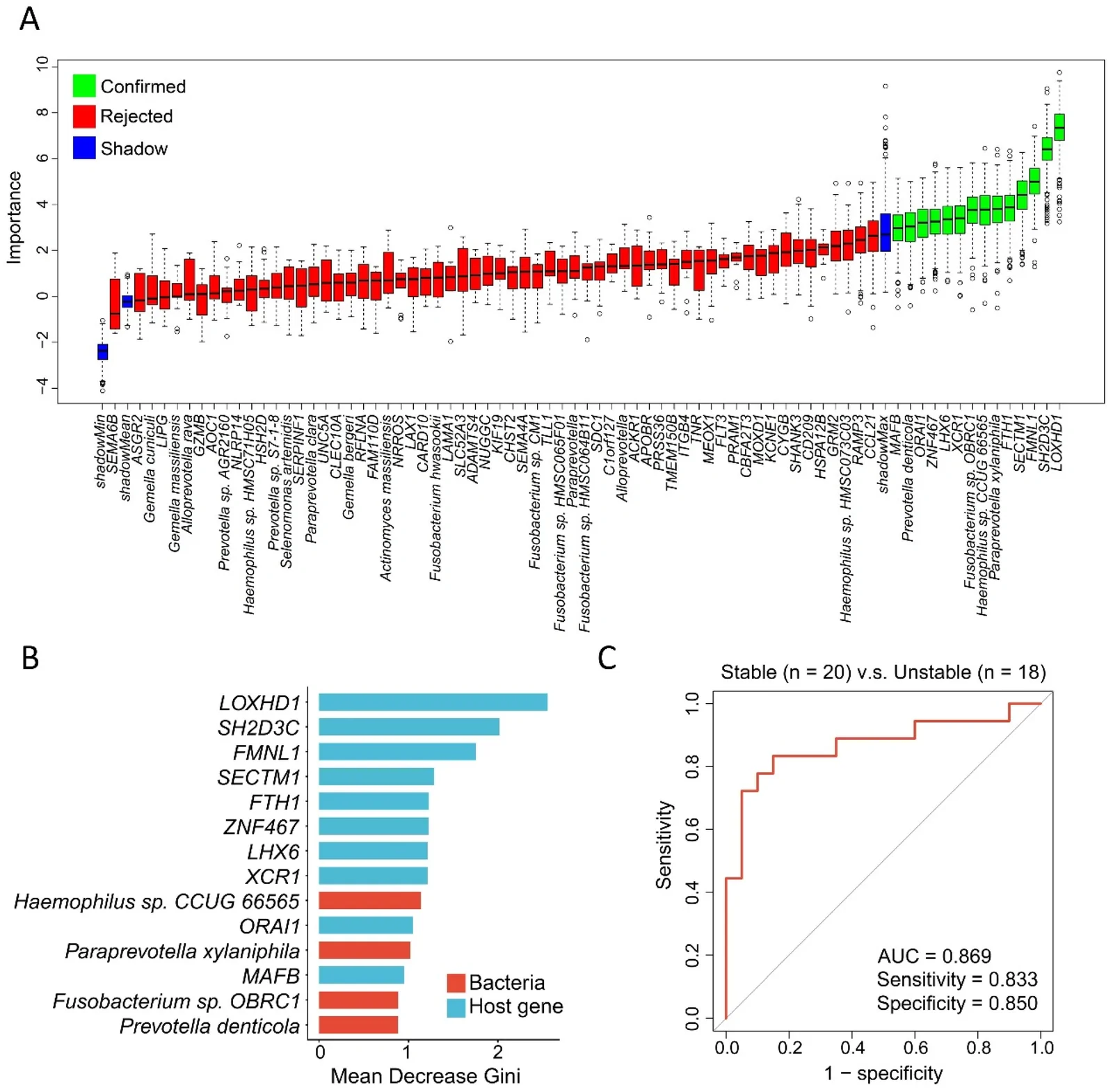
Integrated feature analysis identifies candidate biomarkers of plaque vulnerability. (**A**) The Boruta algorithm iteratively compares the importance of each feature against randomly permuted shadow attributes (shadowMin, shadowMean, shadowMax; blue). Features whose importance significantly exceeds that of the maximum shadow feature are classified as confirmed (green); those that do not are rejected (red). Fourteen features (10 host genes and 4 bacterial taxa) were confirmed as informative discriminators between stable and unstable plaques. (**B**) Feature importance ranking of the 14 Boruta-confirmed biomarkers based on the MDG impurity from the random forest classifier. Blue bars indicate host genes; red bars indicate bacterial taxa. (**C**) ROC curve demonstrating the collective discriminatory capacity of the 14-feature panel in the integrated cohort (stable plaque, n = 20; unstable plaque, n = 18). AUC, area under the curve; CI, confidence interval; MDG, mean decrease in Gini; ROC, receiver operating characteristic.

To prioritize discriminative candidate biomarkers from our integrated host–microbe dataset, we employed the Boruta feature selection algorithm. This machine learning approach systematically evaluated the classification importance of all 54 host DEGs and 21 decontaminated, unstable plaque-enriched bacterial taxa. The Boruta analysis isolated 14 core features, comprising 10 host genes and 4 bacterial taxa, whose predictive importance exceeded that of randomized shadow variables (Figure 6A). Ranking these 14 confirmed features by their mean decrease in Gini (MDG) index revealed that host transcriptomic changes were the primary features contributing to classification, with the genes *LOXHD1*, *SH2D3C*, and *FMNL1* emerging as the top three discriminators between stable and unstable plaques (Figure 6B). Importantly, key microbial taxa were also retained within the top ranks, specifically *Haemophilus* sp. *CCUG 66565*, *Paraprevotella xylaniphila*, *Fusobacterium* sp. *OBRC1*, and *Prevotella denticola*. The retention of both host remodeling/inflammatory genes and specific mucocutaneous pathobionts reinforces the concept that plaque destabilization involves coordinated host–microbiome co-variation.

**Figure 7:**
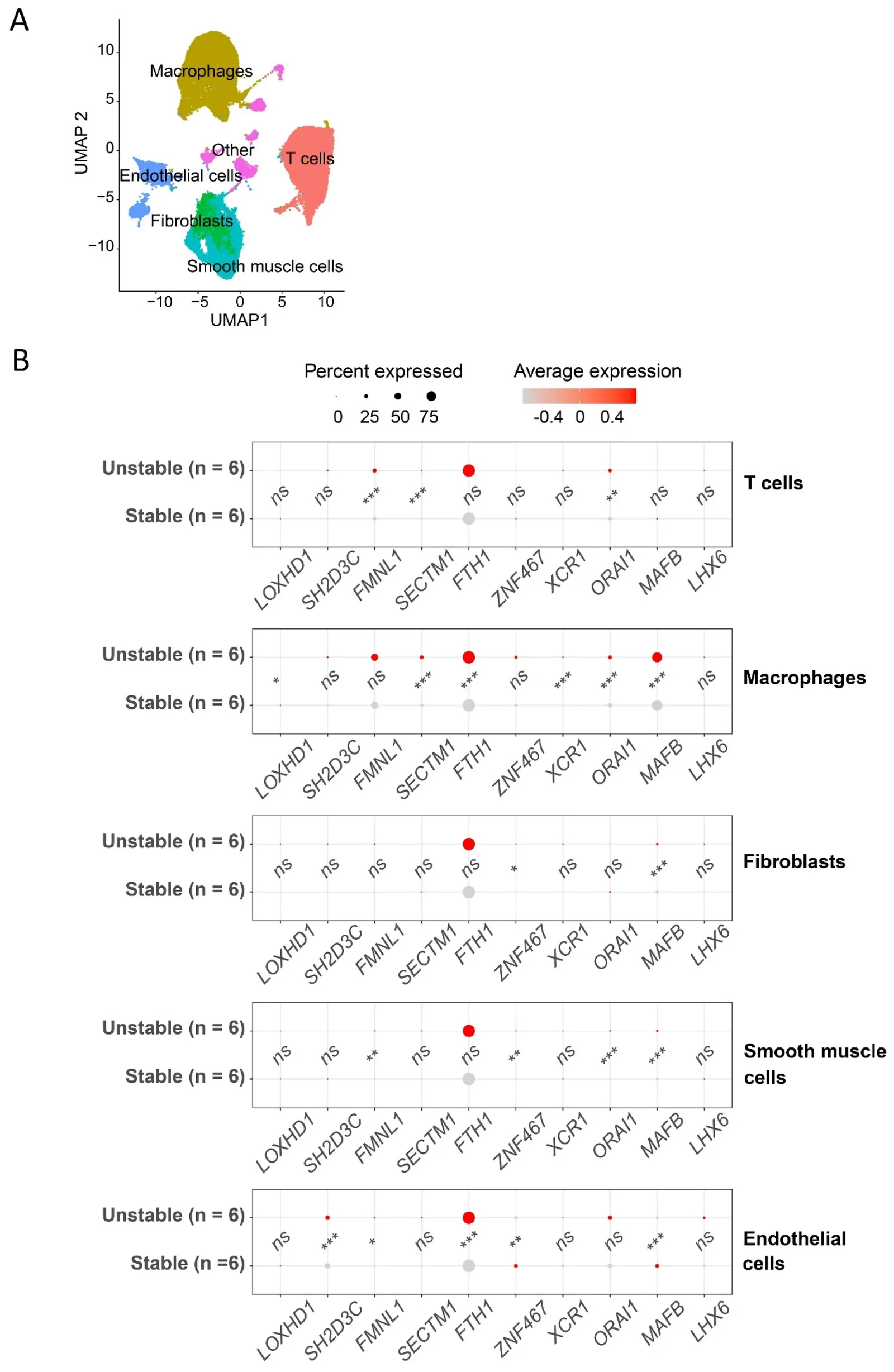
Single-cell transcriptomic profiling reveals cell-type-specific differential gene expression between unstable and stable conditions. (**A**) UMAP embedding of integrated single-cell RNA sequencing data. Cells are colored and annotated by five identified major cell lineages: macrophages, T cells, smooth muscle cells, fibroblasts, and endothelial cells. (**B**) Cell-type-specific dot plots illustrating the expression patterns of a selected panel of 10 target genes across five major cell populations. Within each cell type panel, gene expression is compared between unstable (n = 6) and stable (n = 6) groups. The size of the dot represents the percentage of cells expressing the respective gene within that specific group. The color intensity of the dot reflects the scaled average expression level, with red indicating higher expression and gray indicating lower expression. Statistical significance of differential expression between unstable and stable groups was calculated using the Wilcoxon rank-sum test. * *p* < 0.05, ** *p* < 0.01, *** *p* < 0.001; ns, not significant. UMAP, uniform manifold approximation and projection.

To evaluate the classification performance and quantify the collective diagnostic capacity of this selected 14-feature panel, we constructed a random forest classifier utilizing 5-fold cross-validation to distinguish stable (n = 20) from unstable plaques (n = 18). The integrated cross-kingdom model demonstrated strong discriminatory capacity, achieving an AUC of 0.869 (95% CI: 0.744–0.995) (Figure 6C), although this combined diagnostic model is presented strictly as an exploratory framework. To address the necessity of independent validation and to elucidate the specific cellular origins of these diagnostic targets, we evaluated the host genes from our panel using an independent single-cell RNA-seq dataset of human carotid plaques (GSE253903). Expression analysis at single-cell resolution confirmed that key host genes were significantly upregulated in the unstable plaque microenvironment, exhibiting highly distinct, cell-type-specific dynamics. Notably, *FMNL1* was significantly upregulated specifically within T cells, smooth muscle cells, and endothelial cells of unstable plaques. *SH2D3C* showed significant upregulation localized predominantly to endothelial cells, while *FTH1* displayed marked enrichment across macrophages and endothelial cells in the unstable group. Importantly, this validation revealed that the bulk-level transcriptomic shifts associated with vulnerability are driven by localized, cell-specific activities rather than universal upregulation across all cell types (Figure 7). Altogether, this computational evidence highlights the discriminatory power of the combined host–microbe panel, suggesting that integrating intraplaque microbial abundance with host transcriptomics holds significant promise for evaluating and diagnosing plaque instability in future prospective validations.

## 4. Discussion

This integrative transcriptomic and microbial profiling study provides a comprehensive, hypothesis-generating multi-omics framework for understanding stage-associated host–microbiome co-variation during carotid atherosclerotic plaque progression. Our principal findings are as follows. First, intraplaque microbial communities undergo systematic compositional reorganization during plaque progression, characterized by preserved alpha diversity but marked beta diversity divergence. Second, this microbial remodeling coincides with the coordinated, step-wise upregulation of 54 host genes implicated in ECM degradation, immune chemotaxis, and PI3K/Akt signaling. Third, causal mediation analysis using an unsupervised WGCNA-derived module eigengene (ME2) demonstrated significant direct associations of specific microbial taxa with plaque vulnerability, highlighting a dynamic coupling between microbiome infiltration and host transcriptomic deterioration. Fourth, an integrated 14-feature biomarker panel demonstrated strong, albeit exploratory, cross-validated discriminatory capacity for identifying plaque vulnerability (AUC = 0.869).

The dissociation between preserved alpha diversity and significant beta diversity divergence across disease stages indicates that carotid plaque progression is accompanied by community-level compositional reorganization rather than a simple expansion or contraction of microbial richness. This pattern is consistent with ecological models of dysbiosis, wherein pathological states are characterized by altered community configuration rather than reduced diversity per se [18], mirroring findings in other chronic inflammatory conditions such as inflammatory bowel disease [19] and periodontitis [20]. Crucially, while our stringent three-tier decontamination strategy and external 16S cross-referencing lend confidence to these observations, the detection of microbial sequences from low-biomass human RNA-seq data remains technically challenging. It is inherently susceptible to environmental contamination, mapping artifacts, and sequencing noise. Therefore, the retained microbial signatures must be interpreted strictly as hypothesis-generating signals rather than definitive proof of in vivo colonization.

The taxonomy-based computational prediction of KEGG functional profiling of the 21 bacterial taxa significantly enriched in unstable plaques (dominated by classical oral and gut pathobionts) outlines a speculative yet biologically plausible pathogenic program. Specifically, the predicted upregulation of programmed cell death pathways (e.g., “apoptosis,” “ferroptosis”), immunological shifts (e.g., “IL-17 signaling,” “Th17 cell differentiation”), and hypoxia-driven inflammation (“HIF-1 signaling”) aligns with established mechanisms of advanced plaque vulnerability. These findings suggest that specific unstable plaque-associated bacteria may potentially correlate with necrotic core expansion, immune hyperactivation, and local lipid dysregulation [21,22,23,24,25,26,27]. Notably, it is imperative to emphasize that these inferred KEGG pathways are generated from taxonomy-based computational predictions rather than direct metagenomic or metatranscriptomic measurements. Therefore, discussions of “apoptosis,” “ferroptosis,” “IL-17 signaling,” “Th17 cell differentiation,” and “HIF-1 signaling” herein must be interpreted with caution, strictly as predicted functional potential rather than as demonstrated biological activity. While these functional profiles require future validation, they pinpoint targeted pathways for subsequent experimental interventions.

**Figure 8:**
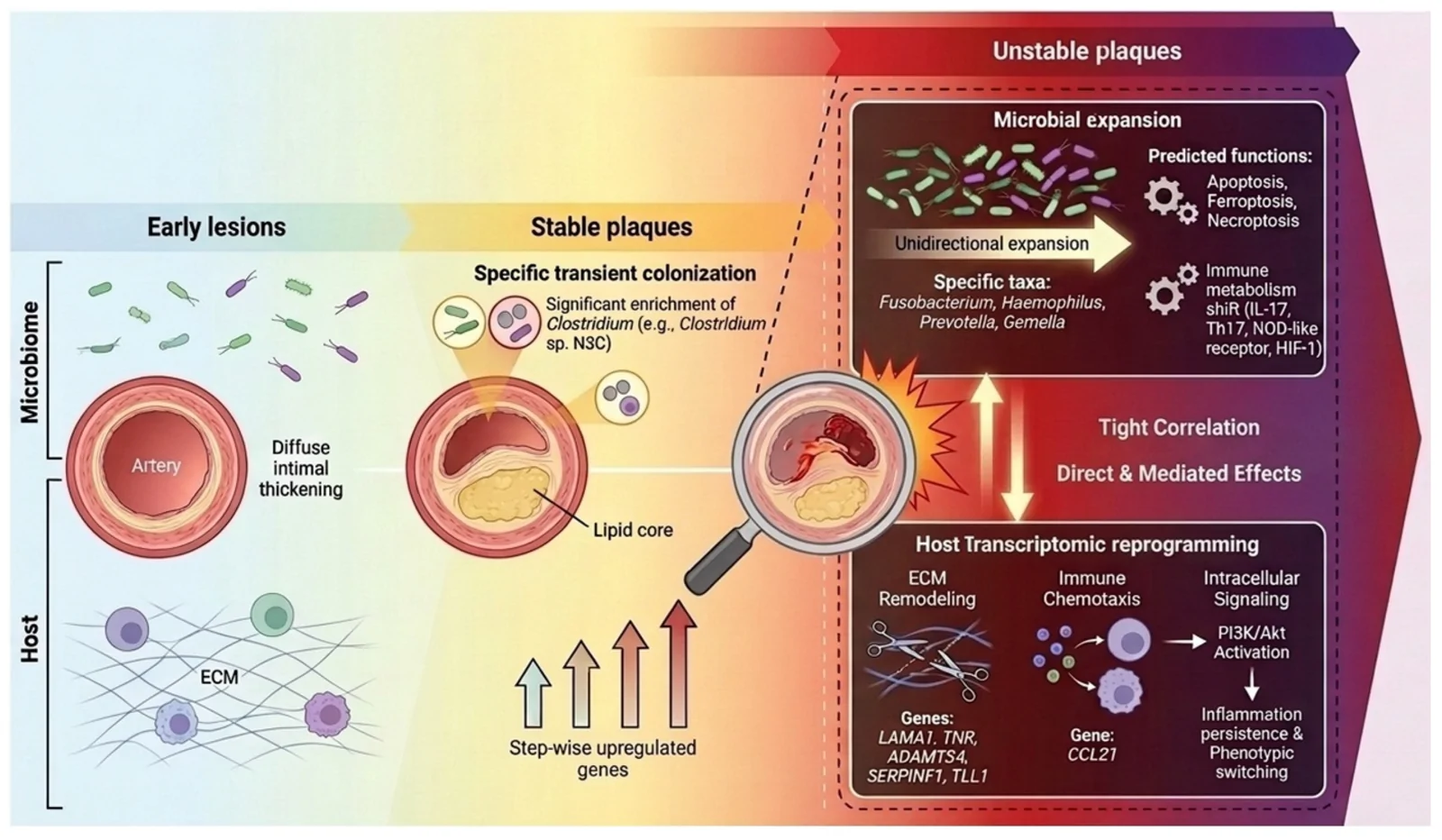
Progressive intraplaque microbial dynamic shifts synergize with host matrisome remodeling and immune chemotaxis to drive atherosclerosis destabilization. The proposed mechanism highlights the bidirectional interplay between intraplaque microbiota and host responses during atherosclerosis progression. While stable plaques exhibit transient specific microbial colonization (e.g., *Clostridium*), unstable plaques are characterized by a unidirectional expansion of pathogenic taxa (e.g., *Fusobacterium*, *Haemophilus*, *Prevotella*, and *Gemella*) associated with cell death and immune metabolism shifts. This microbial expansion exhibits tight correlations and direct/mediated effects with step-wise upregulated host genes. Synergistically, these cross-kingdom interactions drive profound host transcriptomic reprogramming, specifically promoting ECM remodeling (via *LAMA1*, *TNR*, *ADAMTS4*, *SERPINF1*, and *TLL1*), *CCL21*-mediated immune chemotaxis, and PI3K/Akt-driven persistent inflammation, which collectively precipitate plaque rupture.

During the initial stages of plaque formation, microbial abundance remained relatively stable. However, at the stable-to-unstable transition, distinct interactive patterns emerged. Under our microbiome mediation model, while the indirect mediation effect (ACME) did not reach FDR-corrected significance, likely due to the statistical power constraints (73.91%) inherent in the sample size of advanced lesions, the direct effects (ADE) remained statistically significant for key taxa. Rather than viewing host and microbial factors as operating in isolation, this persistent association suggests that microbial tissue infiltration is dynamically coupled with host transcriptomic destabilization. This stage-associated broadening of microbial signals provides suggestive evidence that the intraplaque microbiota may play a more prominent and potentially synergistic role during the critical window of advanced plaque destabilization. Mechanistically, as proposed in our integrated biological model (Figure 8), these microorganisms might be involved in compromising plaque structural integrity through secreted proteases, lipopolysaccharides, or bioactive metabolites [28,29].

A particularly noteworthy aspect of our study is the construction of a cross-kingdom machine learning model. Boruta-based feature prioritization, performed prior to cross-validation folds using the full dataset, identified a 14-feature candidate biomarker panel that discriminated stable from unstable plaques. Although the model achieved an encouraging AUC of 0.869, we explicitly acknowledge that developing a classifier using a relatively small number of samples while evaluating numerous candidate features increases the risk of model overfitting and potential data leakage. Consequently, the reported AUC may be overestimated, and this combined diagnostic model is presented strictly as an exploratory framework serving as a proof of concept for dual-omics diagnostic tools.

Our findings advance prior intraplaque microbiota studies [4,5,30] by simultaneously profiling host and microbial signals from the same RNA-seq libraries, effectively eliminating cross-platform biases. Concurrently, our host transcriptomic findings corroborate the critical roles of ECM remodeling, immune chemotaxis [15,16,31,32], and PI3K/Akt signaling [17,33] in late-stage carotid plaque evolution.

Several limitations of the present study must be acknowledged. First, as this is a cross-sectional analysis of public datasets, the results show associations rather than direct causal effects; causal mediation inferences herein cannot definitively establish causality. Furthermore, this study relies exclusively on computational and in silico analyses; the absence of strictly controlled in vitro or in vivo “wet-lab” experiments limits our ability to mechanically validate the precise biological functions of the identified host–microbe interactions. Second, extracting microbial signals using PathSeq is less sensitive than dedicated physical extraction methods (the gold standard for low-biomass research), reinforcing the hypothesis-generating nature of our ecologically suggestive signals. Third, taxa such as *Clostridium* and *Neisseria* encompass environmental species, raising the possibility of transient bacteremia or sub-threshold contamination, while taxa like *Haemophilus* may reflect barrier dysfunction-driven translocation rather than primary pathogenesis. Fourth, integrating three independent GEO datasets introduces inherent clinical heterogeneity, including variations in patient demographics, sequencing platforms, and tissue processing procedures, which may introduce unmeasured confounding variables. Fifth, due to the cross-sectional nature of the datasets, we are unable to track dynamic morphological progression; consequently, it remains unknown how many plaques, with or without microbial dysbiosis, actually demonstrated longitudinal morphological progression. Sixth, because our study relies on retrospective bulk tissue profiling without spatial mapping, we cannot confirm whether the morphologically determined vulnerability and the host transcriptomic profiles correspond perfectly to the exact regions of hemodynamic instability or altered blood flow. Additionally, although we successfully leveraged single-cell RNA-seq data to validate host genes, standard single-cell transcriptomic platforms (which rely on poly-A capture) fundamentally fail to capture unpolyadenylated bacterial RNA. This technical barrier precluded us from validating the exact cellular localization of intraplaque microbes or exploring host–microbiome co-variation at true single-cell resolution. Seventh, while patient baseline characteristics are compiled in Table S1, exact clinical annotations regarding whether the disease was clinically stable or unstable (e.g., the specific incidence of TIA, acute stroke, or other cardiovascular events) were not fully reported by the original depositors for all cases. Finally, although the host targets of our diagnostic panel were biologically substantiated at the single-cell level, the complete 14-feature cross-kingdom machine learning model currently lacks an independent external bulk RNA-seq cohort to verify its external predictive accuracy (AUC). Consequently, the integrated diagnostic model remains strictly an exploratory proof of concept that requires rigorous evaluation in future large-scale, independent, multi-center prospective cohorts.

Future research should prioritize: (1) orthogonal validation of key microbial findings using specific qPCR, FISH, or cultivation-based methods; (2) functional characterization via in vivo colonization models; (3) experimental investigation of candidate microbial-derived metabolites; and (4) large-scale, multi-center validation of the proposed diagnostic panel.

## 5. Conclusions

In summary, this integrative dual-omics analysis provides suggestive evidence of stage-associated co-variation between host transcriptomic programs and intraplaque microbial communities during carotid atherosclerosis progression. We successfully identified a composite 14-feature cross-kingdom panel that demonstrates exploratory diagnostic capacity for plaque vulnerability. The striking contrast between the stable microbiome during early plaque formation and the significant microbial expansion during the transition to unstable states indicates that the intraplaque microbiota is associated with late-stage plaque destabilization. Ultimately, this study provides a critical, hypothesis-generating multi-omics foundation, offering fresh mechanistic insights and candidate biomarkers that will drive the future development of stage-tailored, microbiome-aware diagnostic strategies for cardiovascular disease.

## Data Availability

All datasets analyzed in this study are publicly available. Carotid plaque RNA-seq datasets (GSE120521, GSE198600, GSE104140, GSE253903, and GSE308470) were obtained from the NCBI Gene Expression Omnibus (GEO; https://www.ncbi.nlm.nih.gov/geo). The processed expression matrices and microbial abundance profiles generated during this study are available from the corresponding author upon request.

## Supplementary Materials

The following supporting information can be downloaded at https://www.mdpi.com/article/10.3390/biomedicines14081708/s1, Table S1: Technical specifications and detailed clinical characteristics of the integrated RNA-sequencing datasets; Table S2: Sequencing read counts and bacterial recovery rates; Table S3: Results of statistical mediation analysis for selected bacterial species; Table S4: Sensitivity analysis comparing the identified genes and bacterial taxa between the merged datasets and GSE104140 alone.

## Abbreviations

ACME	Average causal mediation effect
ADE	Average direct effect
AUC	Area under the curve
BWA	Burrows–Wheeler Aligner
CI	Confidence interval
DEG	Differentially expressed gene
ECM	Extracellular matrix
FDR	False discovery rate
FISH	Fluorescence in situ hybridization
GEO	Gene Expression Omnibus
GO	Gene Ontology
BP	Biological Process
GSVA	Gene set variation analysis
IQR	Interquartile range
KEGG	Kyoto Encyclopedia of Genes and Genomes
KO	KEGG Orthology
MDG	Mean decrease in Gini
ME	Module eigengene
PCA	Principal component analysis
PCoA	Principal coordinate analysis
PERMANOVA	Permutational multivariate analysis of variance
RNA-seq	RNA sequencing
ROC	Receiver operating characteristic
SEM	Standard error of the mean
TIA	Transient ischemic attack
TMAO	Trimethylamine N-oxide
TMM	Trimmed mean of M-values
TOM	Topological overlap matrix
WGCNA	Weighted gene co-expression network analysis
